# Structure-activity relationship of amino acid analogs to probe the binding pocket of sodium-coupled neutral amino acid transporter SNAT2

**DOI:** 10.1007/s00726-024-03424-3

**Published:** 2024-10-19

**Authors:** Sebastian Jakobsen, Maria Pedersen, Carsten Uhd Nielsen

**Affiliations:** Department of Physics, Chemistry and Pharmacy, University of Southern Denmark, Campusvej 55, Odense M, DK-5230 Denmark

**Keywords:** SNAT2, Amino acid transporter, Structure-activity relationship, Amino acid analogs, PC-3, FMP assay

## Abstract

The sodium-coupled neutral amino acid transporter SNAT2 (SLC38A2) has been shown to have important physiological functions and is implicated in various diseases like cancer. However, few compounds targeting this transporter have been identified and little is known about the structural requirements for SNAT2 binding. In this study, the aim was to establish the basic structure-activity relationship for SNAT2 using amino acid analogs. These analogs were first studied for their ability to inhibit SNAT2-mediated ^3^H-glycine uptake in hyperosmotically treated PC-3 cells. Then to identify substrates a FLIPR membrane potential assay and o-phthalaldehyde derivatization of intracellular amino with subsequent quantification using HPLC-Fl was used. The results showed that ester derivatives of the C-terminus maintained SNAT2 affinity, suggesting that the negative charge was less important. On the other hand, the positive charge at the N-terminus of the substrate and the ability to donate at least two hydrogen bonds to the binding site appeared important for SNAT2 recognition of the amine. Side chain charged amino acids generally had no affinity for SNAT2, but their non-charged derivatives were able to inhibit SNAT2-mediated ^3^H-glycine uptake, while also showing that amino acids of a notable length still had affinity for SNAT2. Several amino acid analogs appeared to be novel substrates of SNAT2, while γ-benzyl L-glutamate seemed to be inefficiently translocated by SNAT2. Elaborating on this structure could lead to the discovery of non-translocated inhibitors of SNAT2. Thus, the present study provides valuable insights into the basic structural binding requirements for SNAT2 and can aid the future discovery of compounds that target SNAT2.

**Supplementary Information:**

The online version contains supplementary material available at 10.1007/s00726-024-03424-3.

## Introduction

Amino acids (AAs) play various important roles in cell function and growth. Consequently, amino acid transporters (AATs) have gained interest for their involvement in health and disease (as reviewed in (Jakobsen and Nielsen 2024; Kandasamy et al. 2018)). The human genome encodes more than 60 AATs despite their limited substrate pool of 20 proteinogenic AAs and a few non-proteinogenic AAs. This redundancy underscores their vital role in cell homeostasis. AATs often adopt similar structural folds as exemplified by the LeuT-fold commonly seen in the Amino Acid-Polyamine-Organocation (APC) transporter family (Edwards et al. 2018). Despite their general commonalities in both structure and the substrates they transport, AATs are intricately unique in their transport mechanism (uniport, symport, antiport), the electrochemical gradients they utilize, and their exact substrate specificity which can vary from both a broad recognition of most AAs to a narrower specificity that only accepts a single AA.

To understand the unique characteristics of each AAT the discovery of chemical research tools, such as substrates and inhibitors, and solving their 3D protein structures are needed. Despite some progress, only a few AATs have experimentally solved 3D structures and an array of chemical tools to study them (Jakobsen and Nielsen 2024). Many others, such as the sodium-coupled neutral amino acid transporter SNAT2 (SLC38A2), remain largely underexplored. SNAT2, a member of the APC transporter family, preferentially transports small to medium-sized neutral amino acids through symport with sodium ions and has a ubiquitous tissue expression (Sugawara et al. 2000; Yao et al. 2000). It is upregulated by several stimuli, including amino acid starvation, hyperosmotic stress, insulin, and glucagon (Gazzola et al. 2001; Kashiwagi et al. 2009; Ortiz et al. 2011). Thus, SNAT2 facilitates the cellular uptake of AAs during fed (following insulin secretion) and fasted states, along with maintaining cell volume through intracellular accumulation of organic osmolytes under hyperosmotic conditions. It is also indicated that SNAT2 functions as a transceptor (a contraction of “transporter” and “receptor”), meaning that it similarly to a receptor can convey signals to the cell when compounds associate with the transporter (Pinilla et al. 2011).

SNAT2 has gained interest for its involvement in e.g. placental nutrient transport (Vaughan et al. 2021), alveolar fluid clearance (Weidenfeld et al. 2021), pancreatic function (Zhang et al. 2023), and cancers like gastric cancer and breast cancer (Morotti et al. 2021; Zhu et al. 2023). Despite its prominent role in health and disease, SNAT2 still lacks a solved 3D protein structure and proper chemical probes to further its study. Historically the N-methylated amino acid analog MeAIB (α-(methylamino)isobutyric acid) has been used to inhibit and study the SLC38 transporters, but it doesn’t discriminate between the different system A SLC38 members and is also transported by PAT1 (SLC36A1) (Chen et al. 2003; Christensen et al. 1965). Recently, a novel and potent non-amino acid inhibitor of SNAT2 was proposed (Gauthier-Coles et al. 2022), however, the observed inhibition of the compound has been unable to be recreated (Jakobsen et al. 2023).

Our study aims to advance the discovery of chemical tools for SNAT2 research and uncover the structural requirements for SNAT2 binding, enhancing our understanding of its substrate specificity. Thus, through uptake studies and FLIPR membrane potential assays in hyperosmotically treated PC-3 cells, known to upregulate SNAT2 (Nielsen et al. 2022), we have performed a SNAT2-based structure-activity-relationship (SAR) study using amino acid analogs and uncovered novel SNAT2 substrates.

## Materials and methods

### Materials

[2-^3^H]-Glycine (45.2 Ci/mmol), Ultima GoldTM scintillation fluid, and scintillation vials (6 mL, Pony VialTM) were from Perkin Elmer (Waltham, MA, USA). Dulbecco’s Modified Eagle Medium/Nutrient Mixture F-12 (DMEM/F12), penicillin/streptomycin (100x), L-glutamine (200 mM), sodium pyruvate (100 mM), L-ascorbic acid, phosphate-buffered saline (PBS), trypsin-EDTA (10x) all suitable for cell culture where from Sigma Aldrich (Merck KGaA, Darmstadt, Germany). Fetal Bovine Serum (FBS) for cell culture was from Biowest (Nuaillé, France). Hanks Balanced Salt Solution (HBSS, Gibco), L-alaninol (98%), glycinamide hydrochloride (98%), L-pyroglutamic acid (98%), L-azetidine-2-carboxylic acid (99+%), L-homoserine (99%), N(ε)-carbobenzyloxy-L-lysine (98%), N(α)-carbobenzyloxy-L-glutamine (99%), and γ-benzyl L-glutamate (99%) were from Thermo Fischer Scientific (Waltham, MA, USA). N-Formyl-DL-alanine (≥ 98.0%), pyrrole-2-carboxylic acid (≥ 98.0%), L-alanine ethyl ester hydrochloride (≥ 98.0%), L-alanine benzyl ester hydrochloride (≥ 98.0%), N,N-dimethylglycine hydrochloride (≥ 98.0%), O-benzyl-L-serine (≥ 99.0%), N(ε)-acetyl-L-lysine (≥ 98.0%), L-norvaline (≥ 99.0%), L-norleucine (≥ 99.0%), trans-4-hydroxy-L-proline (≥ 99.0%), Oxfenicine (≥ 99.0%), 3-cyclohexyl-L-alanine (97%), L-pipecolinic acid (97%), β-Benzyl L-aspartate (≥ 98.0%), methanol (≥ 99.9%, HPLC suitable), and acetonitrile (99.99%, HPLC suitable) were from VWR (Radnor, PA, USA). Sodium bicarbonate solution (7.5%), 4-(2-hydroxyethyl)-1-piperazineethanesulfonic acid (HEPES) (≥ 99.5%), D-(+)-raffinose pentahydrate (≥ 98.0%), Triton-X 100, L-glutamine (≥ 99.5%), L-asparagine (≥ 99.5%), L-alanine (≥ 99.5%), L-proline (≥ 99.0%), L-serine (≥ 99%), L-threonine (≥ 98%), L-valine (≥ 98%), L-glutamic acid (≥ 99%), L-lysine monohydrochloride (≥ 98%), L-phenylalanine (≥ 98%), L-arginine hydrochloride (≥ 98%), D-alanine (≥ 98%), D-asparagine (99%), D-glutamine (≥ 98%), D-valine (≥ 98%), L-alanine methyl ester hydrochloride (99%), L-alanine tert-butyl ester hydrochloride (≥ 99.0%), picolinic acid (≥ 99%), sodium L-lactate (≥ 99.0%), glycine (98%), sarcosine (98%), betaine (≥ 98%), taurine (≥ 99%), monopotassium phosphate (≥ 99.0%), 2-mercaptoethanol (≥ 99.0%), phthaldialdehyde (OPA) reagent (product nr. P0532), and dimethyl sulfoxide (DMSO) were from Sigma Aldrich (Merck KGaA, Darmstadt, Germany). 3-Aminobutan-2-one hydrochloride (95%) and 2-amino-N-benzylacetamide (98%) were from BLDpharm (Shanghai, China).

### Cell culture

PC-3 cells (ECACC 90,112,714) were obtained from the European Collection of Authenticated Cell Cultures (ECACC; UK Health Security Agency, Salisbury, UK) and were received in passage 31. PC-3 cells were maintained in DMEM/F12 supplemented with penicillin (100 U ⋅ mL^− 1^), streptomycin (0.1 mg ⋅ mL^− 1^), L-glutamine (2 mM), sodium pyruvate (2 mM), L-ascorbic acid (20 µg ⋅ mL^− 1^), and 10% fetal bovine serum (FBS). The cells were kept in an incubator at 37 °C in an atmosphere of 5% CO_2_ and with 94–97% relative humidity and the culture medium was changed every 2–3 days. For uptake studies, the PC-3 cells were seeded in 24-well plates (area 1.9 cm^2^) with a density of 1.5 ⋅ 10^5^ cells ⋅ cm^− 2^ two days before the experiment. For CellTiter-Glo and FLIPR membrane potential (FMP) assays PC-3 cells were seeded in 96-well plates (0.32 cm^2^) at a density of 1.5 ⋅ 10^5^ cells ⋅ cm^− 2^ two days before the experiment. For hyperosmotic stimulation, PC-3 cells were incubated in hyperosmotic media 24 h before the experiment. The hyperosmotic media was prepared by supplementing normal isoosmotic culture medium with 200 mM raffinose to reach an osmolality of approximately 500 mOsm ⋅ kg^− 1^ (512 ± 13 mOsm ⋅ kg^− 1^). Experiments were performed on PC-3 cells in passages 2–15 after thawing.

### Radiolabeled uptake studies

^3^H-glycine uptake in hyperosmotically treated PC-3 cells was performed as previously described (Jakobsen et al. 2023). 5-minute uptake of 0.5 µCi ⋅ mL^− 1 3^H-glycine (11.1 nM) was used, and each uptake was normalized to the uptake obtained in a control situation (31.2 ± 5.7 fmol ⋅ cm^− 2^ ⋅ min^− 1^) without subtracting the background uptake (2.62 ± 0.67 fmol ⋅ cm^− 2^ ⋅ min^− 1^). Compounds that inhibited uptake by more than 20% at the highest tested concentrations were tested in the CellTiter-Glo viability assay (Promega, Madison, WI, USA), to rule out that the observed inhibition was not caused by reduced cell viability (Fig. S1 in Supplementary Information SI).

### FLIPR membrane potential (FMP) assay

PC-3 cells exposed to hyperosmotic media for 24 h were used to identify SNAT2 substrates in the FMP assay. The media was aspirated, and the cells were preincubated in HBSS + for 10 min at 37 °C and 220 rpm. Then the buffer was aspirated, and cells were then incubated in 50 µL HBSS + containing 0.55 mg ⋅ mL^− 1^ (1x) FMP probe (Blue component A from Molecular Devices, San Jose, CA, USA) for 30 min at 37 °C and 220 rpm. The plate was then placed in a CLARIOstar^®^ Plus plate reader from BMG LABTECH (Ortenberg, Germany) at 37 °C and subsequently measured the fluorescence (Ex. 530 nm, Em. 565 nm) of each well one column at a time. First, the baseline was measured for 30 s by measuring each well every 3 s. The plate was then removed from the reader and 50 µL of substrate solutions containing 0.55 mg ⋅ mL^− 1^ FMP probe was added. These solutions were prepared by mixing equal parts solution containing 2x FMP probe and solutions containing 4x the final concentration of substrate. After the addition of the substrate solution the plate was returned to the reader and shaken at 200 rpm for 5 s and then fluorescence intensity was measured for 120 s. Following this, the baseline of the next column was measured and followed the same procedure as just described.

### Quantification of intracellular amino acids and analogs using OPA derivatization followed by HPLC-Fl analysis

Derivatization of AAs and analogs using o-phthalaldehyde (OPA) and 2-mercaptoethanol (2-ME) to make fluorescent derivatives was used to measure the intracellular amounts of AAs and analogs following uptake in hyperosmotically treated PC-3 cells. Before starting the experiment, the medium was aspirated, and the cells were preincubated in HBSS + for 15 min at 37 °C and 220 rpm. The cells were then incubated with 500 µL HBSS+ (background) or HBSS + containing AAs or analogs at given concentrations for 30 min at 37 °C and 220 rpm. After aspirating the donor solutions, the cells were washed thrice with 500 µL of ice-cold PBS while keeping the cells on ice. 200 µL of ice-cold 1x-trypsin was added to each well and allowed to distribute for 20–30 min while keeping the cells on ice. The plate was then transferred to an incubating microplate shaker at 37 °C and 220 rpm for about 8 min or until the cells had detached. 300 µL of ice-cold PBS was added to each well to resuspend the cells and transfer them to centrifuge tubes. The cells were centrifuged at 1000 G for 5 min (4 °C), the supernatant was removed, the cells were resuspended in 300 µL ice-cold PBS, and then centrifuged again at 1000 G for 5 min (4 °C). After removing the supernatant, the cell pellet was lysed by adding 100 µL of 80% methanol (20% ultrapure water) and placing the tubes in an ultrasonic bath for 10 min. The tubes were then centrifuged at 15,000 G for 15 min (4 °C) and the resulting supernatant was used for analysis.

The OPA reagent was prepared by spiking with 2 µL 2-ME per mL of OPA regent and was considered stable for use within one day. 10 µL of the cell samples or standards containing a mixture of the AAs and analogs of interest were placed in HPLC vials with 100 µL inserts. All samples were analyzed using a Prominence UFLC system (LC-20AD pumps, RF-20 A XS fluorescence detector, Nexera SIL-30AC autosampler, and a CTO-10AS VP column oven) from Shimadzu (Kyoto, Japan) using a modified version of the following application note (Shimadzu 2014). The autosampler kept the samples at 10 °C and the pretreatment function was used to automatically mix the samples with 10 µL of the OPA reagent and wait 4 min before loading 2 µL onto a YMC-Triart C18 column (75 × 3.0 mm, 1.9 μm, Mikrolab Aarhus, Viby J, Denmark). The fluorescent derivatives were separated at a flow rate of 0.5 mL ⋅ min^− 1^ and column temperature of 35 °C with mobile phase A (20 mM KH_2_PO_4_ in ultrapure water, pH 6.90) and mobile phase B (45:40:15% MeCN: MeOH: H_2_O) using the following gradient (as % of mobile phase B); 15% for 0–4 min, 30% for 4.1–11 min, 35% for 11.5–14.5 min, 60% for 15–20.5 min, 75% for 21–22.5 min, 90% for 22.6–24.5 min, and 15% for 24.6–25 min. The fluorescence detector was set to excite at 350 nm and detect at 450 nm.

The resulting chromatograms were integrated and the area under the curve (AUC) of the standard samples was used to generate standard curves to determine the concentration of AAs and analogs in the cell samples. The pooled standard curves and example chromatograms can be seen in the SI (Figs. S2, S3). Amino acid levels detected in the background cell samples were subtracted from the other cell samples. L-Alanine benzyl ester only generated a peak with an equal retention time as L-alanine. It was assessed that the addition of OPA reagent increased the hydrolysis of the ester, thus only creating the L-alanine OPA derivative. To ensure that the observed result was not because of L-alanine benzyl ester hydrolyzing before the addition of the OPA reagent, its stability was confirmed in aqueous solution over 3 days through HPLC-UV analysis (SI, Fig. S4). Thus, the L-alanine peak was used to measure the presence of L-alanine benzyl ester in samples.

### Data analysis

For concentration-dependent inhibition studies GraphPad Prism 10.1.2 was used to fit the data to the “[Inhibitor] vs. response (three parameters)”-model:

$$Y\; = \;Bottom\; + \;\frac{{Top - Bottom}}{{1\; + \;\left( {\frac{X}{{I{C_{50}}}}} \right)}}$$

Where *Y* is the % uptake, *X* is the concentration of the inhibitor, Top and Bottom are the top and bottom plateau respectively, and IC_50_ is the half maximal inhibitory concentration. These studies were performed in at least 3 independent cell passages (*n* = 3) and a curve was defined for each independent passage. However, for each compound, the top and bottom were constrained to be the same amongst the different cell passages. The top and bottom values ranged between 113 − 92% and 3.1–10% respectively. For L-alanine tert-butyl ester and N, N-dimethylglycine full inhibition was not achieved so the bottom value was constrained to be equal to the bottom value of similar compounds.

For the FMP data, the baseline was defined as the mean fluorescence of the first data point before substrate addition. The baseline of each well was then subtracted from the data point to get the ΔF. Plotting ΔF as a function of time and calculating the area under the curve (AUC) after substrate addition was used as a measure of SNAT2 activity. For concentration-dependent studies, the EC_50_ was determined by fitting to the following model using GraphPad Prism 10.1.2:

$$Y\; = \;Bottom\; + \;X\; \cdot \frac{{Top - Bottom}}{{E{C_{50}}\; + \;X}}$$

Where *Y* is the AUC and *X* is the concentration of the compound.

### Statistical analysis

All values are represented as means ± standard error of the mean (SEM) unless otherwise stated. IC_50_s and EC_50_s had their means and SEM calculated using the log-transformed values. GraphPad Prism 10.1.2 was used to detect statistically significant differences in the data. One-way or two-way ANOVAs were performed followed by Dunnett’s or Šidák’s multiple comparisons test, respectively.

## Results & discussion

### Inhibition of SNAT2 mediated 3H-glycine uptake in PC-3 cells

To study SNAT2-mediated transport hyperosmotically treated PC-3 prostate cells were used, as previous work showed that SNAT2 was upregulated and the main carrier of glycine, sarcosine, and L-proline (Nielsen et al. 2022). siRNA knockout of SNAT2 and inhibition by betaine (shown to be selective for SNAT2 over SNAT1 (Nishimura et al. 2014) reduced ^3^H-glycine uptake by around 66% and 86% respectively, thus making ^3^H-glycine uptake in hyperosmotically treated PC-3 cells a good model for SNAT2 transport (Jakobsen et al. 2023; Nielsen et al. 2022).

#### Amino acid analogs with C- and N-terminal modifications

**Fig. 1:**
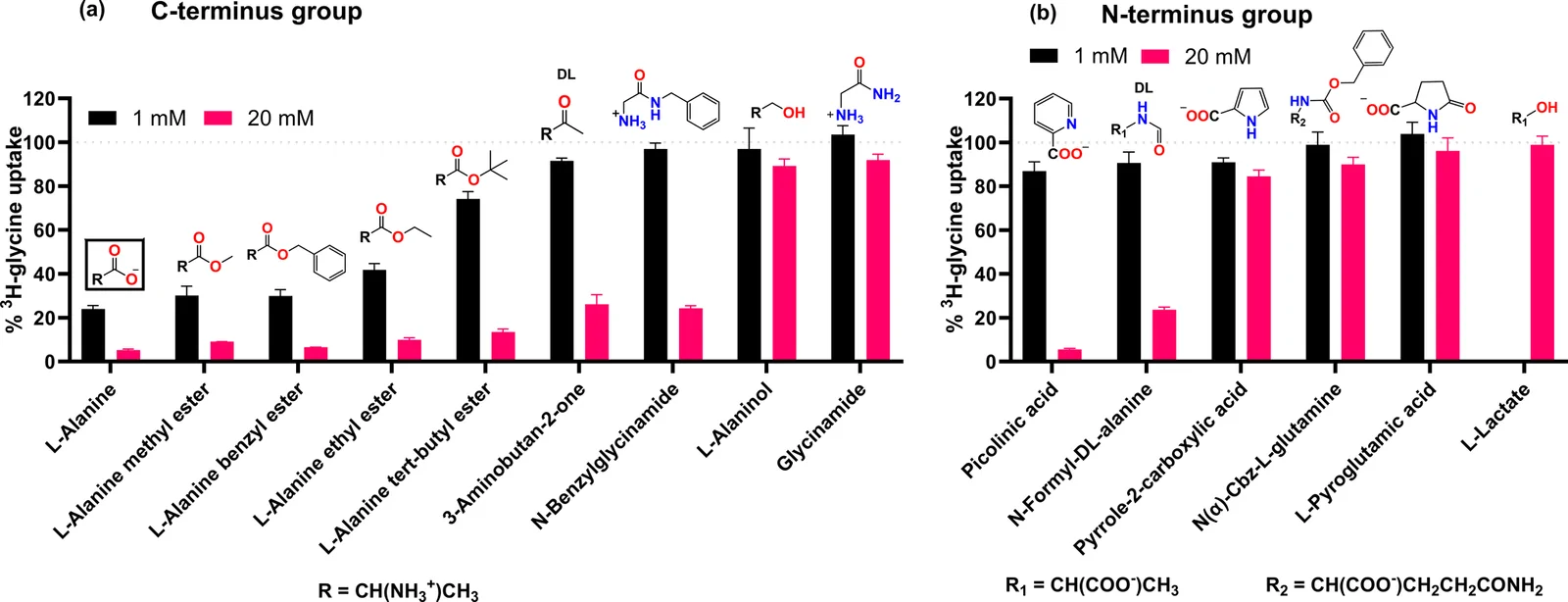
Normalized ^3^H-glycine uptake in hyperosmotically treated PC-3 cells in the presence of 1 or 20 mM of various AAs and analogs with modifications to **(a)** the -COOH group or **(b)** the -NH_2_ group. The structures of the modified residues are shown and natural proteinogenic AAs are framed. Generally, L-isomers were used, but racemic mixtures are indicated by “DL”. All experiments were performed using 10 mM HEPES buffer in HBSS, pH 7.4. The cells were exposed to 0.5 µCi ⋅ mL^− 1 3^H-glycine (11.1 nM) for 5 min at 37 °C. Values are reported as means ± SEM for three independent cell passages (*n* = 3)

A selection of AA analogs was used to assess the impact of carboxylic acid (C-terminus) and amine (N-terminus) modifications on the recognition of AAs by SNAT2. The carboxylic acid and amine of natural AAs are charged at pH 7.4, and modifications to these groups can help identify the importance of such charges for SNAT2 recognition. For the C-terminus group, it is evident that various esters of L-alanine still retain their ability to inhibit SNAT2-mediated ^3^H-glycine uptake (Fig. 1a). This indicates that the negative charge of the COO^−^ group is not detrimental for recognition. However, when the carboxylic group is replaced with an alcohol (L-alaninol) or amide (glycinamide) group it appears that potency is lost. The ketone derivative of DL-alanine (3-aminobutan-2-one) retains some affinity for SNAT2 despite being a racemic mixture of D- and L-isomers. Hence, the carbonyl group of the -COOH functionality appears to be more important than the hydroxyl group regarding SNAT2 affinity. In a similar study performed for LAT1 (SLC7A5), another AAT member of the APC family, leucine analogs also revealed that esters of the C-terminus maintain affinity, while the alcohol analog lost affinity (Nagamori et al. 2016). Cryo-EM structures of LAT1-ligand complexes have revealed that the carboxylic acid oxygens interact with the LAT1 protein backbone as hydrogen bond acceptors (Yan et al. 2021), and it is possible that SNAT2 recognizes the AA carboxyl group in a similar way.

Comparing the different L-alanine esters it is generally seen for the aliphatic esters, that increasing the size of the alkyl ester group decreases the affinity of the AA analog. Interestingly, the benzyl ester retains a similar affinity as the methyl ester, despite the increase in size. However, it is important to remember that spatially the benzyl group is flat and able to participate in other types of interactions with the SNAT2 protein. L-alanine along with the different ester derivatives were subject to concentration-dependent inhibition studies to determine their IC_50_ values (Table 1, Fig. S5 in SI for concentration-inhibition curves). These IC_50_ values reflect the same tendency in potency as seen in Fig. 1a while also showing that L-alanine has the best affinity for SNAT2 of the compounds tested.

Looking at the N-terminus modifications (Fig. 1b), limited inhibition is seen for the analogs, which could be linked to none of them being positively charged at this position like regular AAs. Most of these analogs have poor inhibition of ^3^H-glycine uptake even at 20 mM except picolinic acid and N-formyl-DL-alanine. However, it should be noted that concentration-dependent inhibition studies revealed that picolinic acid has a very steep IC_50_ curve (Fig. S6 in SI), which indicates an effect not necessarily caused by direct SNAT2 inhibition. This trend of N-terminus modifications abolishing affinity is also seen in the LAT1 SAR study, again reflecting a similarity between the two transporters in their general recognition of the amino acid backbone (Nagamori et al. 2016). In Cryo-EM structures of LAT1, the amino acid amine is recognized through hydrogen bonds and a π-cation interaction with Phe252, thus highlighting why the positive charge appears to be important for recognition by LAT1 (Yan et al. 2021). Overall, it seems that affinity is dictated more by the presence of a positive charge at the -NH_2_ position than a negative charge at the -COOH position.

**Table 1:** IC_50_ values of select AAs and analogs derived from concentration-dependent inhibition studies of SNAT2 mediated ^3^H-glycine uptake in hyperosmotically treated PC-3 cells. Mean and SEM values were calculated using -log transformed values (pIC_50_) and the number of independent cell passages (n) is reported in the table

Compound	IC_50_, mM	pIC_50_ ± SEM	*n*
L-Alanine	0.17	[3.8 ± 0.079]	3
L-Homoserine	0.25	[3.6 ± 0.025]	3
L-Alanine methyl ester	0.33	[3.5 ± 0.10]	3
L-Alanine benzyl ester	0.39	[3.4 ± 0.021]	3
L-Alanine ethyl ester	0.42	[3.4 ± 0.097]	3
O-Benzyl-L-serine	0.44	[3.4 ± 0.037]	3
L-Serine	0.46	[3.3 ± 0.067]	3
Glycine	0.52	[3.3 ± 0.11]	3
3-Cyclohexyl-L-alanine	0.63	[3.2 ± 0.021]	3
L-Proline	0.64	[3.2 ± 0.020]	4
L-Azetidine-2-carboxylic acid	0.66	[3.2 ± 0.027]	4
γ-Benzyl L-glutamate	0.68	[3.2 ± 0.047]	3
Sarcosine	0.71	[3.1 ± 0.027]	3
L-Pipecolinic acid	2.9	[2.5 ± 0.032]	3
trans-4-Hydroxy-L-proline	3.5	[2.5 ± 0.035]	4
L-Alanine tert-butyl ester	4.1	[2.4 ± 0.092]	3
Betaine	6.6	[2.2 ± 0.037]	3
N,N-Dimethylglycine	23	[1.6 ± 0.012]	3

**Fig. 2:**
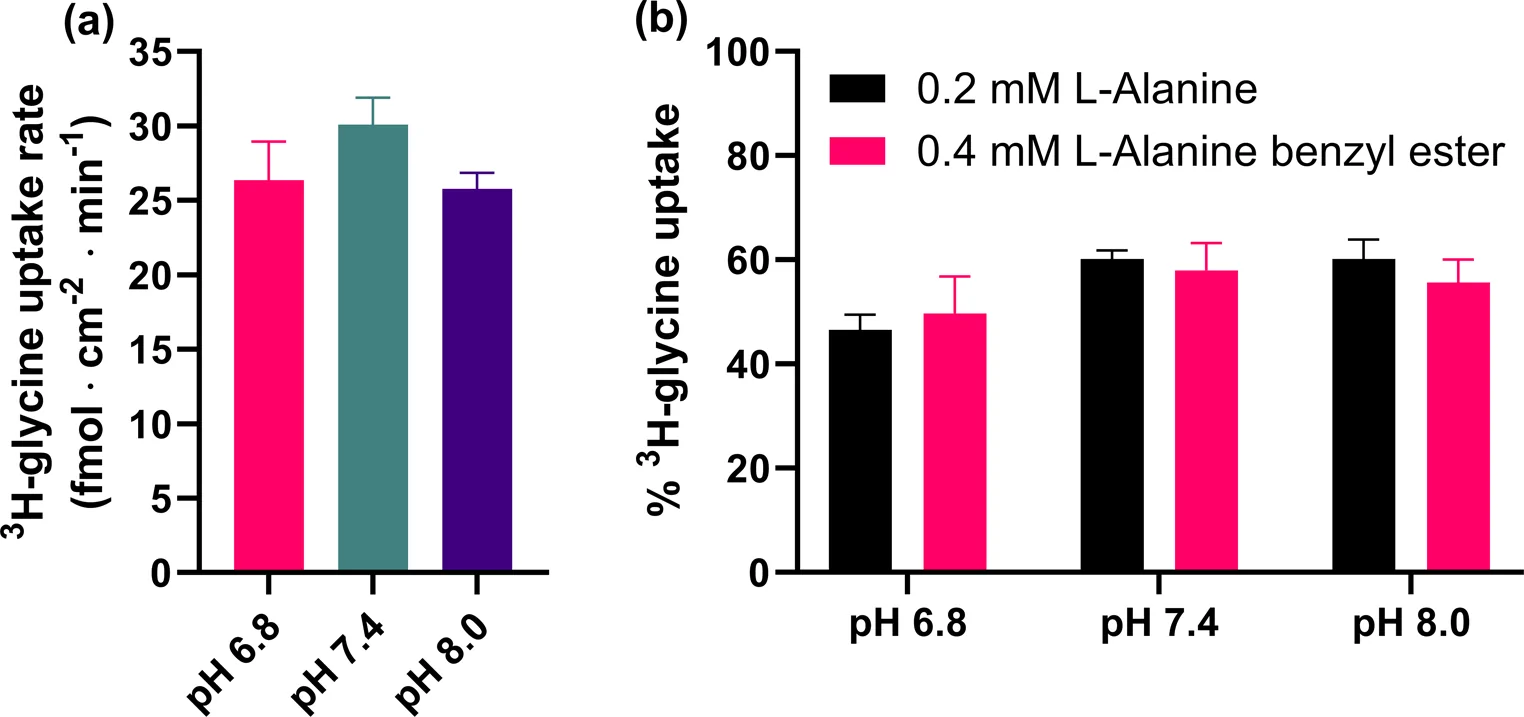
^3^H-glycine uptake in hyperosmotically treated PC-3 cells at pH 6.8, 7.4, or 8.0 in the **(a)** absence or **(b)** presence of 0.4 mM L-alanine benzyl ester or 0.2 mM L-alanine. All experiments were performed using 10 mM HEPES buffer in HBSS at 37 °C. The cells were then exposed to 0.5 µCi ⋅ mL^− 1 3^H-glycine (11.1 nM) for 5 min at 37 °C. Values are reported as means ± SEM for three independent cell passages (*n* = 3). **(a)** One-way ANOVA revealed no statistically significant differences. **(b)** Uptake was normalized to the corresponding control uptake at the same pH level. Two-way ANOVA revealed no statistically significant differences

When the -COOH group is derivatized to an ester or an amide, this also influences the pK_a_ of the amine, making it less basic. The carboxy or carboxamide group has electron-withdrawing effects on the amine, decreasing its basicity. In regular AAs, this effect is counterbalanced by the incentive to form zwitterions, which are overall electroneutral in charge, thus increasing the basicity of the amine. For these C-terminus derivatives, only the electron-withdrawing effect dominates. Using Chem3D^®^ (Version 22.0.0.22) it was predicted that the basic pK_a_’s of the alanine esters and glycinamide were in the range of 7.26–7.37, thus suggesting that the amino group of these analogs are not fully protonated (and charged) at pH 7.4. If the protonated form has better affinity as suggested by the results from the -NH_2_ group analogs, then lowering the pH of the uptake buffer should increase their inhibition of SNAT2. To test this hypothesis L-alanine benzyl ester (pK_a_(NH_3_^+^) = 7.26) inhibition of ^3^H-glycine was tested at pH 6.8, 7.4, and 8.0 and normalized to appropriate buffer controls. L-Alanine (pK_a_(NH_3_^+^) = 10.2) was also tested as a negative control, as its zwitterionic state should be the major species in this pH range. Both compounds were tested at a concentration close to their IC_50_ values (Table 1) to help capture any changes in inhibitory potency. Control experiments revealed no significant differences in the uptake at the different pH levels (Fig. 2a). Interestingly, this doesn’t reflect the pH sensitivity attributed to SNAT2, where increased extracellular pH also increased transport activity (Hatanaka et al. 2000). The relatively narrow pH range was chosen since the PC-3 cell line has been shown to be highly sensitive to pH changes, which could reflect the decrease in apparent uptake at pH 8.0 (Nielsen et al. 2022). In the case of L-alanine benzyl ester and L-alanine (Fig. 2b) a two-way ANOVA revealed no significant differences in inhibition at the different pH levels. It seems that inhibition is greatest at pH 6.8 but this tendency is seen for both compounds and thus does not seem to be related to the protonation state of the amine. This hints that it might not be the positive charge of the amine that is important for SNAT2 recognition but possibly the presence of at least two hydrogen bond donors. Looking at the analogs in Fig. 1b it is seen that the derivatives not only lack a positive charge but also only contain at most one hydrogen able to participate in hydrogen bonds at the amine position. This hypothesis will be elaborated on in the next section. It should also be noted that increasing the length between the C- and N-terminus was unfavorable, as β- and γ-AAs showed poor SNAT2 inhibition compared to α-AAs (data not shown).

#### N-methylated amino acid analogs and L- vs. D-isomers

**Fig. 3:**
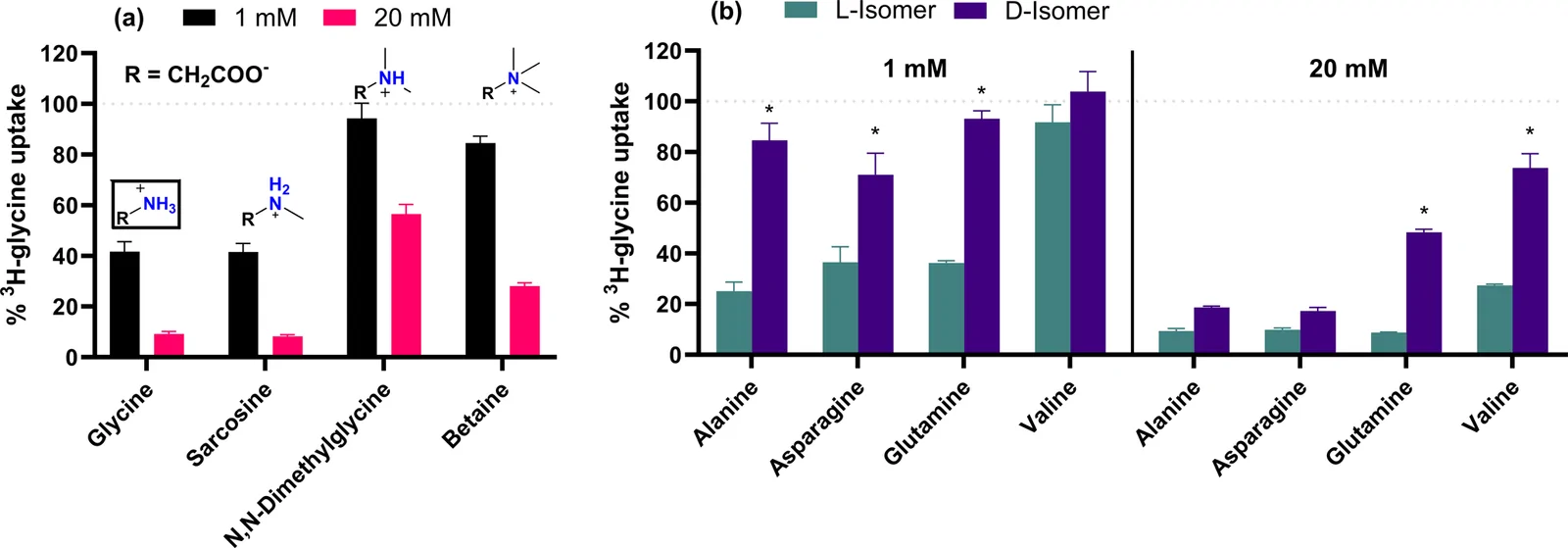
Normalized ^3^H-glycine uptake in hyperosmotically treated PC-3 cells in the presence of 1 or 20 mM of **(a)** glycine or its methylated analogs or **(b)** the L and D-isomers of alanine, glutamine, asparagine, and valine. All experiments were performed using 10 mM HEPES buffer in HBSS, pH 7.4. The cells were exposed to 0.5 µCi ⋅ mL^− 1 3^H-glycine (11.1 nM) for 5 min at 37 °C. Values are reported as means ± SEM for three independent cell passages (*n* = 3) except for 1 mM L- and D-glutamine which is performed in triplicates in a single cell passage (*N* = 3). **(b)** Statistically significant differences of the D-isomer compared to the L-isomer detected by two-way ANOVA are shown (*: *p* < 0.05)

Since the system A SNAT transporters were first characterized by their ability to recognize N-methylated AAs (Christensen et al. 1965) it seemed natural to explore the impact of N-methylation on SNAT2 affinity. Figure 3a shows a series of glycine derivatives with different degrees of N-methylation and their inhibition of SNAT2-mediated ^3^H-glycine uptake. These results show that glycine and the monomethylated analog sarcosine have the greatest potency, with similar inhibition of SNAT2 uptake at 1 or 20 mM. Increasing the degree of methylation appears to weaken potency, however, the trimethylated analog betaine appears to have a better affinity for SNAT2 than the dimethylated N,N-dimethylglycine, and also shows an apparent selectivity for SNAT2 over SNAT1 (Nishimura et al. 2014). The distinct change in affinity seen for N,N-dimethylglycine also supports the idea that the presence of two hydrogen bond donors at this position is important for SNAT2 recognition, as hypothesized above. Cryo-EM structures of the APC members LAT1 and b^0,+^AT (SLC7A9) also show intricate hydrogen bonding networks with AA amine hydrogens, suggesting a common feature for recognition of amino acids within this family (Yan et al. 2020, 2021).

It is seen that SNAT2 generally prefers the L-isomers of AAs to that of D-isomers as shown in Fig. 3b. However, the D-isomers still retain some degree of affinity, so that at 20 mM both the L- and D-isomers of alanine and asparagine show full inhibition of ^3^H-glycine uptake. Interestingly, it appears that the discrepancy in affinity between L- and D-asparagine is smaller than that of L- and D-glutamine, which might be because of the increased length of the side chain in glutamine.

#### Amino acid analogs with side chain modifications

**Fig. 4:**
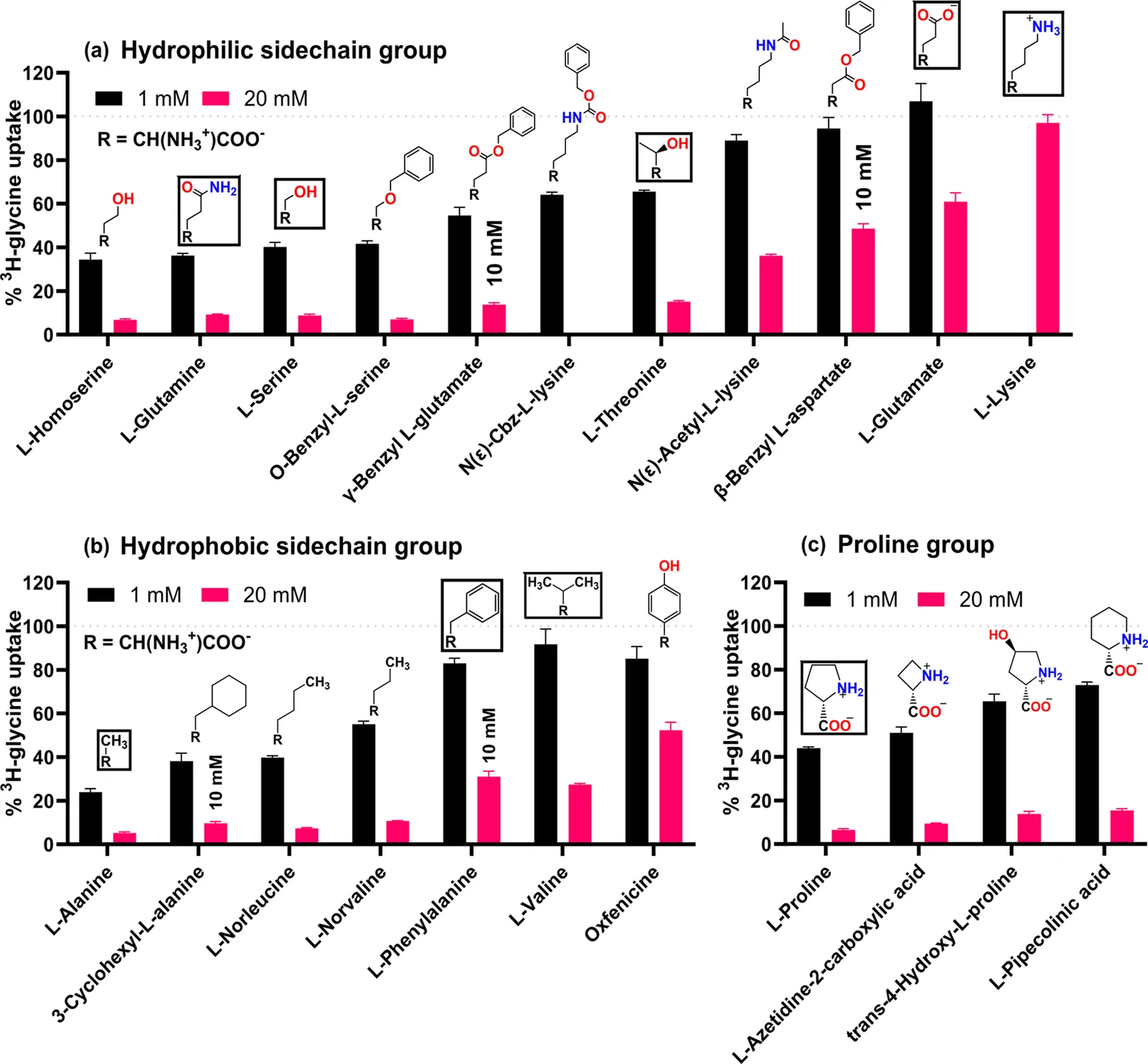
Normalized ^3^H-glycine uptake in hyperosmotically treated PC-3 cells in the presence of 1 or 20 mM (or 10 mM when indicated) of various AAs and analogs with side chain modifications of **(a)** hydrophilic AAs or **(b)** hydrophobic AAs or **(c)** L-proline analogs. The structures of the modified residues are shown and natural proteinogenic AAs are framed. All experiments were performed using 10 mM HEPES buffer in HBSS, pH 7.4. The cells were exposed to 0.5 µCi ⋅ mL^− 1 3^H-Glycine (11.1 nM) for 5 min at 37 °C. Values are reported as means ± SEM for three independent cell passages (*n* = 3), except for 20 mM L-glutamine (*n* = 16) and 1 mM L-glutamine, L-threonine, L-glutamate, and L-lysine, which was performed in triplicates in a single cell passage (*N* = 3)

Various amino acid analogs with modifications at the side chain were explored to help understand the substrate selectivity of SNAT2. These analogs were separated into analogs of hydrophilic AAs, hydrophobic AAs, and analogs of L-proline and their inhibition of SNAT2-mediated uptake can be seen in Fig. 4. As for natural AAs, SNAT2 typically prefers smaller neutral AAs such as L-alanine and L-serine or medium-sized hydrophilic AAs such as L-glutamine (Hatanaka et al. 2000). Charged AAs such as L-glutamate and L-lysine have limited affinity as is also reflected in Fig. 4a. Interestingly, derivatization of these charged AAs that removes their charged functionality appears to increase their affinity as seen for γ-benzyl-L-glutamate, Nε-cbz-L-lysine, and Nε-acetyl-L-lysine. Nε-Cbz-L-lysine indicates that there should be space for a long side chain in the SNAT2 binding pocket. Unfortunately, this compound could not be tested at higher concentrations due to solubility limitations. Interestingly, β-benzyl L-aspartate appears to have a poorer affinity for SNAT2 when compared to the other benzyl derivatives. The aromatic ring is 5 bond lengths from the α-carbon which appears to be unfavorable compared to the 3 or 6 bond-length distance seen in O-benzyl-L-serine and γ-benzyl-L-glutamate respectively. However, it is generally seen that the introduction of benzyl groups to the hydrophilic AAs maintains affinity or leads to increased affinity in the case of charged AAs. This is interesting considering that L-phenylalanine and oxfenicine are relatively poor at inhibiting SNAT2 when looking at the hydrophobic side chain group (Fig. 4b). Even more interesting is that the non-aromatic analog of L-phenylalanine, 3-cyclohexyl-L-alanine, appears to have greater affinity than L-phenylalanine.

It seems that branching at the β-carbon negatively impacts affinity as evidenced by the difference in inhibition between L-valine and L-norvaline. L-Threonine also appears to inhibit less than L-serine and thus corroborates this hypothesis. L-Threonine seems to have a better SNAT2 affinity than L-valine, suggesting that a hydrophilic group is favorable at this position, possibly due to hydrogen bonding or a hydrophilic environment.

For the proline group (Fig. 4c), L-proline appears to have the greatest affinity with the 4-membered ring analog L-azetidine-2-carboxylic acid retaining a similar affinity. However, the introduction of a trans-configured hydroxyl group at the 4 position or increasing the ring size to 6 members, seems to have a noteworthy negative impact on affinity. This again suggests that increasing the bulk volume too close to the α-carbon appears to be less favorable, whereas a bulkier group such as a benzyl group can be introduced 4 bond lengths away from the α-carbon without an apparent affinity penalty.

### SNAT2 substrate identification using the FLIPR membrane potential (FMP) assay

Given the diversity of the tested AA analogs, we wanted to investigate if these analogs were translocated by SNAT2, thus exploring the structural requirements for substrate translocation. To investigate if the analogs were substrates of SNAT2, their ability to induce transient membrane depolarization due to SNAT2-mediated co-transport of Na^+^ was investigated using the FLIPR membrane potential (FMP) assay. A SNAT2 substrate leads to an influx of Na^+^ and thus transiently depolarizes the membrane which leads to an increase in fluorescence in the FMP assay (as seen for SNAT2 in (Gauthier-Coles et al. 2022). To our knowledge, this is the first time the FMP assay is used with PC-3 cells. Hence, to validate the assay in the PC-3 cell model, concentration-dependent studies, using the SNAT2 substrate L-alanine in both hyperosmotically and isoosmotically treated PC-3 cells, were performed. Taurine was included as a negative control since it has been shown not to influence SNAT2 uptake in PC-3 cells (Nielsen et al. 2022). Taurine itself is a substrate for both the proton-coupled PAT1 and sodium and chloride-coupled TauT (Nielsen et al. 2017). Hence taurine itself will not affect the membrane potential as PAT1 is not expressed in PC-3 cells (Nielsen et al. 2022) and TauT is a low-capacity carrier (Nielsen et al. 2017). L-alanine is able to increase the fluorescence intensity from the baseline (ΔF) in both isoosmotically and hyperosmotically treated PC-3 cells in a concentration-dependent and saturable fashion, while 20 mM taurine did not lead to an increase in fluorescence (Fig. S7 in SI). Generally, it is seen that ΔF is higher for the hyperosmotically treated PC-3 cells compared to the isoosmotically treated cells. However, for the isoosmotic condition, a higher baseline fluorescence is seen (Fig. S8 in SI), which could be related to the higher number of cells with a larger volume compared to hyperosmotically treated cells that enter cell cycle arrest. Using the AUCs from the kinetic fluorescence curves and plotting them as a function of L-alanine concentrations leads to EC_50_s of 0.14 mM (pEC_50_ = 3.86 ± 0.11) and 0.25 mM (pEC_50_ = 3.61 ± 0.05) for the isoosmotic and hyperosmotic conditions, respectively (Fig S7 in SI). This is similar to the IC_50_ (0.17 mM) determined in the radiolabeled uptake study (Table 1).

A subset of the most potent AA analogs representing the C-terminus and side chain modifications were tested in the FMP assay for their ability to stimulate SNAT2-mediated Na^+^ translocation. 10 mM L-alanine and L-serine were included as positive controls, whereas L-arginine was included as a negative control. Given that L-arginine has a net positive charge, this gave some insight into how cations influence the membrane potential sensitive assay, as these could potentially hyperpolarize the membrane. This is relevant since the L-alanine esters likely contain cationic species. Generally, it is seen that 10 mM L-serine and 10 mM L-alanine achieve similar AUCs, that reflect a saturated system (Table 2). The addition of buffer, 20 mM taurine, or 20 mM L-arginine all have little influence on the fluorescence intensity. Of the tested AA analogs, L-homoserine, L-norleucine, L-alanine methyl ester, 3-cyclohexyl-L-alanine, O-benzyl-L-serine, L-alanine ethyl ester, and L-pipecolinic acid all elicit responses that suggest that these analogs are substrates of SNAT2. L-alanine benzyl ester and γ-benzyl L-glutamate lead to little or no stimulation of SNAT2 activity and could thus potentially be non-translocated inhibitors. However, L-alanine benzyl ester increases the fluorescence signal at 5 mM but not at 10 mM. In the CellTiter-Glo assay, 20 mM L-alanine benzyl ester led to a 23% decrease in cell viability after 10 min of exposure (Fig. S1 in SI). So, these findings may be related to a cell toxic effect or an interaction with the FMP probe at higher concentrations which causes the diminished signal. Interestingly, both of these possible non-translocated analogs are benzyl derivatives, while another benzyl derivative O-benzyl-L-serine appears to be translocated and is a substrate of SNAT2. O-benzyl-L-serine and γ-benzyl L-glutamate are structurally very similar, and it can be hypothesized that the longer distance to the benzyl group in the side chain seen in γ-benzyl L-glutamate is the reason for its inability to be translocated. For instance, it is observed for LAT1 that translocation is more efficient for smaller substrates (Bahrami et al. 2023).

**Table 2:** Stimulation of the FMP assay in hyperosmotically treated PC-3 cells by AAs and analogs. Stimulation of the assay is measured by the area under the curve (AUC) of the resulting fluorescence response-time curve after substrate addition. AUC values are normalized to the response from the addition of 10 mM L-alanine. All values are represented as means ± SEM

Compound	Concentration, mM	AUC (∙ 10^5^), RFU ∙ s	% of 10 mM L-alanine	*n*
Buffer		-1.4 ± 0.1	-6.4 ± 0.5	4
L-Alanine	10	22.1 ± 0.9	100 ± 3.9	4
L-Serine	10	24 ± 0.9	108.6 ± 4.2	3
L-Arginine	20	-0.97 ± 0.3	-4.4 ± 1.2	3
Taurine	20	-1.0 ± 0.2	-4.7 ± 0.7	3
L-Homoserine	10	25.9 ± 1.4	117.5 ± 6.2	3
L-Norleucine	10	24.4 ± 1.3	110.4 ± 6.1	3
L-Alanine methyl ester	10	23.5 ± 0.8	106.6 ± 3.8	3
3-Cyclohexyl-L-alanine	3	21.9 ± 0.5	99.2 ± 2.4	3
O-Benzyl-L-serine	10	21.3 ± 0.4	96.8 ± 1.7	3
L-Alanine ethyl ester	10	21.1 ± 0.6	95.4 ± 2.5	3
L-Pipecolinic acid	10	18.1 ± 0.2	81.9 ± 0.8	3
L-Alanine benzyl ester	5	8.1 ± 1.4	40.8 ± 6.1	3
	10	-1.4 ± 3.2	-6.6 ± 14	3
γ-Benzyl L-glutamate	3	1.7 ± 0.4	7.8 ± 1.9	3

### Quantification of intracellular amino acids and analogs using OPA derivatization

**Fig. 5:**
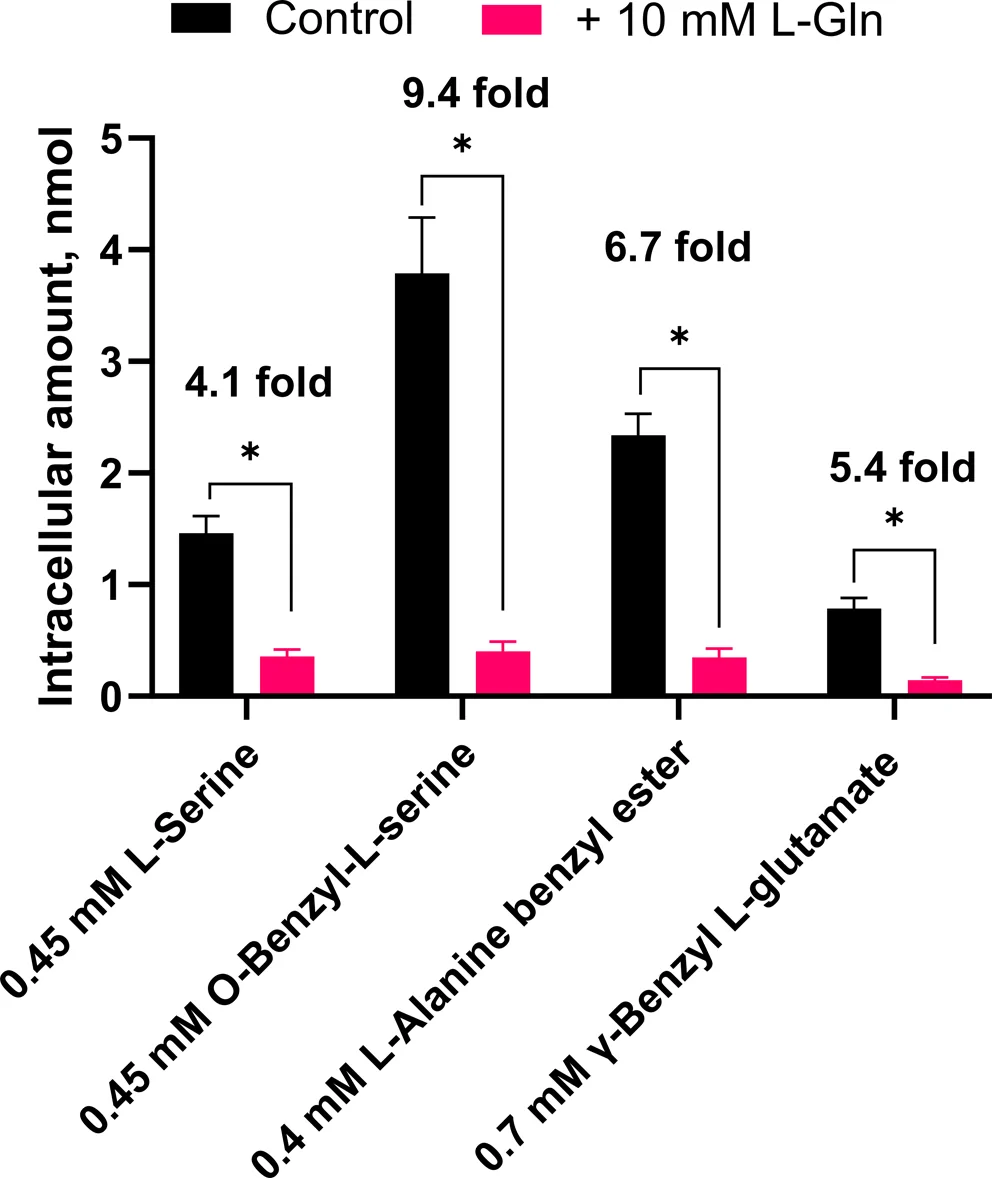
Intracellular accumulation of AAs and analogs in PC-3 cells following 30 min uptake in the presence and absence of 10 mM L-glutamine. All experiments were performed using 10 mM HEPES buffer in HBSS, pH 7.4. The cells were exposed to donor solutions containing the given concentration of AA or analog ± 10 mM L-glutamine for 30 min at 37 °C. Values are reported as means ± SEM for 4 independent cell passages (*n* = 4). Statistically significant differences in intracellular accumulation in the absence or presence of 10 mM L-glutamine detected by two-way ANOVA are shown (*: *p* < 0.05) along with the fold change

To further elaborate on whether the AA analogs were substrates of SNAT2, OPA derivatization was used to quantify the intracellular amounts of AAs and analogs following uptake. AAs with primary amines rapidly form fluorescent compounds when exposed to OPA and 2-mercaptoethanol and can thus be detected through HPLC-FI (Church et al. 1985). To test if L-alanine benzyl ester and γ-benzyl L-glutamate were substrates or non-translocated inhibitors their intracellular amounts were measured following 30 min uptake in the presence or absence of 10 mM L-glutamine. L-serine and O-benzyl-L-serine were also included to represent AA and analog substrates, respectively. Concentrations were chosen based on the IC_50_ values of the individual compounds and the uptake time was increased to 30 min to increase the OPA-derived signal of the substrates being taken up. In Fig. 5 it is seen how the intracellular accumulation of all the tested compounds is significantly decreased in the presence of 10 mM L-glutamine, thus suggesting a SNAT2-mediated entry into the cell. Interestingly, the levels of intracellular accumulation vary between the different AAs and analogs, with O-benzyl-L-serine levels being over double that of L-serine. This could be due to differences in translocation rate or metabolism within the cell. Both L-alanine benzyl ester and γ-benzyl L-glutamate appear to be taken up by SNAT2 since their uptake is inhibited by L-glutamine. Strikingly, γ-benzyl L-glutamate has a relatively low accumulation, which again could be caused by intracellular metabolism but also because of inefficient translocation. It has been shown that γ-benzyl L-glutamate binds to and is likely taken up by the AAT ATB^0,+^ (SLC6A14), which could also be responsible for the observed accumulation in the PC-3 cells (Hatanaka et al. 2004). However, ATB^0,+^ is also electrogenic, and translocation should elicit a response in the FMP assay. Perhaps, the lack of a response in the FMP assay is a sign of inefficient translocation. Overall, this suggests that γ-benzyl L-glutamate binds to SNAT2, but is poorly translocated, possibly making this analog a step towards uncovering a non-translocated inhibitor.

## Conclusion

In conclusion, we have established a structure-activity relationship for the binding of amino acid analogs. It is suggested that only α-amino acids bind to SNAT2 through hydrogen bond acceptor interactions from the carboxylic acid and hydrogen bond donor and possibly cation interactions from the amine. There seems to be some room for side-chain modifications as long as these are without a charge. Several of the tested amino acid analogs were indicated to be novel substrates of SNAT2, while γ-benzyl L-glutamate appears to be inefficiently translocated by SNAT2. These results serve as groundwork for the future development of molecules that target SNAT2.

## Electronic supplementary material

Below is the link to the electronic supplementary material.

Supplementary Material 1

## Data Availability

No datasets were generated or analysed during the current study.
