# Supplementary material for: Structure-activity relationship of amino acid analogs to probe the binding pocket of sodium-coupled neutral amino acid transporter SNAT2
Source: Amino Acids. 2024 Oct 19;56:64. doi: 10.1007/s00726-024-03424-3 (PMC11490426; doi:10.1007/s00726-024-03424-3)
Supplement: Supplementary file 1 [file 726_2024_3424_MOESM1_ESM.docx]

**Supplementary Information**

**Structure-activity relationship of amino acid analogs to probe the binding pocket of sodium-coupled neutral amino acid transporter SNAT2**

Journal: Amino Acids

Sebastian Jakobsen, Maria Pedersen, Carsten Uhd Nielsen*

Department of Physics, Chemistry and Pharmacy, University of Southern Denmark, Campusvej 55, DK-5230 Odense M, Denmark.

*: Correspondence: Carsten Uhd Nielsen, Department of Physics, Chemistry and Pharmacy, University of Southern Denmark, Campusvej 55, DK-5230 Odense M, Denmark. Phone: +45 6550 9427, e-mail: cun@sdu.dk

**Fig. S1** Cell viability of hyperosmotically treated PC-3 cells after 10 min incubation with various amino acids and analogs using the CellTiter-Glo assay. The CellTiter-Glo reagent was prepared following the manufactures instructions. 2.5 mL of the reagent was then mixed with 0.5 mL of HBSS+. PC-3 cells, seeded in white 96-well plates (0.32 cm^2^) and treated with hyperosmotic media 24 hours before the experiment, were used for the viability assay. On the day of the experiment, the cells were preincubated in HBSS+ as described for 15 minutes at 37°C and 220 rpm. The cells were then incubated with compounds for 10 minutes at 37°C and 220 rpm. The solutions were removed and 50 µL of room temperature HBSS+ was added to each well along with 30 µL of the CellTiter-Glo reagent. The plate was placed in a CLARIOstar® Plus plate reader from BMG LABTECH (Ortenberg, Germany) at room temperature and was then shaken for 2 minutes at 220 rpm. Luminescence was measured after 0, 5, 10, and 15 minutes. Background luminescence (obtained from adding CellTiter-Glo reagent to HBSS+ containing wells without cells) was subtracted from the sample luminescence and normalized to control wells only exposed to HBSS+ to determine the % viability. Values are represented as means ± SD for at least 3 replicates in a single cell passage (N≥3). Statistically significant differences from the control detected by one-way ANOVA are shown (*: p < 0.05).

**Fig. S2** Standard curves of L-alanine, L-serine, O-benzyl-L-serine, and γ-benzyl-L-glutamate quantified through OPA derivatization followed by HPLC-FI analysis. The linear regression equations (where y = peak area (mV ⋅min) and x = concentration (µM)) and goodness of fit were y = 533173x + 2554638, R^2^ = 0.9964 (L-alanine); y = 415800x + 157537, R^2^ = 0.9945 (L-serine); y = 379083x + 580969, R^2^ = 0.9875 (O-benzyl-L-serine); and y = 347455x + 441517, R^2^ = 0.9865 (γ-benzyl-L-glutamate). The limits of detection and limits of quantification were determined to be 1.8 µM, 5.4 µM (L-alanine); 2.7 µM, 8.1 µM (L-serine); 4.0 µM, 12 µm (O-benzyl-L-serine); and 4.2 µM, 12.7 µM (γ-benzyl-L-glutamate), respectively. Data points are shown as means ± SD from 4 separate experiments.

**Fig. S3** Representative HPLC-FI chromatograms of amino acid and analog OPA derivatives. The derivative peaks are indicated by their corresponding amino acid. **(a)** Chromatogram of 40 µM standard amino acid and analog mixture. **(b)** Chromatogram of background PC-3 cell lysate sample. **(c)** Chromatogram of PC-3 cell lysate sample following 30-minute uptake of 0.45 mM O-benzyl-L-serine.

**Fig. S4** HPLC-UV chromatograms of L-alanine benzyl ester and benzyl alcohol dissolved in ultrapure water. The samples were analyzed using a Waters 2695 HPLC system connected to a Waters 2487 Dual λ Absorbance Detector (Waters Corporation, MA, USA). The peaks were separated using a Sunfire C18 column (4.6 x 150 mm, 3.5 µm, Waters Corporation, MA, USA) at 45 °C and with a 0.7 ml/min flow of 70% mobile phase A (90/10% H_2_O/MeCN + 0.1% trifluoroacetic acid (TFA)) and 30% mobile phase B (10/90% H_2_O/MeCN + 0.1% TFA). 10 µL samples were injected and the peaks were detected at 254 nm. 1 mM L-alanine benzyl ester and benzyl alcohol had retention times of 2.7 min and 5.0 min respectively, and the peak areas are shown on the chromatograms. **(a)** 1 mM L-alanine benzyl ester at time zero. **(b)** 1 mM L-alanine benzyl ester after 2 hours at room temperature. **(c)** 1 mM L-alanine benzyl ester after 3 days at room temperature. **(d)** 1 mM benzyl alcohol.

**Fig. S5** Concentration-response curves for the inhibition of SNAT2-mediated ^3^H-glycine uptake in hyperosmotically treated PC-3 cells by various amino acids and analogs. Data points are reported as means ± SEM for at least three independent cell passages (n≥3).

**Fig. S6** Concentration-response curve for the inhibition of SNAT2-mediated ^3^H-glycine uptake in hyperosmotically treated PC-3 cells by picolinic acid. The IC_50_ and Hill slope were determined to be 5.6 mM and -4.2, respectively. Data points are reported as means ± SEM for four independent cell passages (n=4).

**Fig. S7** FLIPR membrane potential (FMP) assay in isoosmotically (**a**, **c**) and hyperosmotically (**b**, **d**) treated PC-3 cells. (**a**, **b**) Time-response curve in the development of fluorescence from baseline (ΔF) following the addition of buffer or compound solutions (the gap in the curve reflects the time used to add the solutions). All wells contained 0.55 mg∙mL^-1^ FMP probe. Fluorescence measurements were done 30 sec before compound addition to establish the baseline. Values are represented as means ± SEM for triplicate measurements in a single cell passage (N=3). (**c**, **d**) Concentration-response curve of L-alanine using the area under the curve (AUC) from the FMP time-response curve following compound addition. All values are represented as means ± SEM for three independent cell passages (n=3).

**Fig. S8** FLIPR membrane potential (FMP) assay baseline values for isoosmotically and hyperosmotically treated PC-3 cells. Values represent the mean ± SEM of the baseline across three cell passages (n=3), using 8 replicates for each passage (N=8). A statistically significant difference was detected by an unpaired t-test (p < 0.05).
